# Confounding factors and biases involved in regional differences in the detection rate of thyroid cancer in the second-round Thyroid Ultrasound Examination: the Fukushima Health Management Survey

**DOI:** 10.1093/jrr/rrad044

**Published:** 2023-07-10

**Authors:** Hiroki Shimura, Susumu Yokoya, Satoru Suzuki, Manabu Iwadate, Satoshi Suzuki, Takashi Matsuzuka, Shinichi Suzuki, Fumikazu Hayashi, Masanori Nagao, Tetsuya Ohira, Seiji Yasumura, Hitoshi Ohto, Kenji Kamiya

**Affiliations:** Radiation Medical Science Center for the Fukushima Health Management Survey, Fukushima Medical University, 1 Hikarigaoka, Fukushima City, Fukushima 960-1295, Japan; Department of Laboratory Medicine, Fukushima Medical University Fukushima School of Medicine, 1 Hikarigaoka, Fukushima City, Fukushima 960-1295, Japan; Radiation Medical Science Center for the Fukushima Health Management Survey, Fukushima Medical University, 1 Hikarigaoka, Fukushima City, Fukushima 960-1295, Japan; Thyroid and Endocrine Center, Fukushima Medical University School of Medicine, 1 Hikarigaoka, Fukushima City, Fukushima 960-1295, Japan; Radiation Medical Science Center for the Fukushima Health Management Survey, Fukushima Medical University, 1 Hikarigaoka, Fukushima City, Fukushima 960-1295, Japan; Department of Thyroid and Endocrinology, Fukushima Medical University School of Medicine, 1 Hikarigaoka, Fukushima City, Fukushima 960-1295, Japan; Department of Surgery, Minamisoma Municipal General Hospital, 2-54-6 Takamicho, Haramachi-ku, Minamisoma City, Fukushima 975-0033, Japan; Radiation Medical Science Center for the Fukushima Health Management Survey, Fukushima Medical University, 1 Hikarigaoka, Fukushima City, Fukushima 960-1295, Japan; Department of Thyroid and Endocrinology, Fukushima Medical University School of Medicine, 1 Hikarigaoka, Fukushima City, Fukushima 960-1295, Japan; Radiation Medical Science Center for the Fukushima Health Management Survey, Fukushima Medical University, 1 Hikarigaoka, Fukushima City, Fukushima 960-1295, Japan; Department of Otolaryngology, School of Medicine, Asahi University Hospital, 3-23, Hashimoto-cho, Gifu City, Gifu 500-8523, Japan; Department of Thyroid Therapeutic Surgery, Fukushima Medical University, 1 Hikarigaoka, Fukushima City, Fukushima 960-1295, Japan; Radiation Medical Science Center for the Fukushima Health Management Survey, Fukushima Medical University, 1 Hikarigaoka, Fukushima City, Fukushima 960-1295, Japan; Department of Epidemiology, Fukushima Medical University School of Medicine, 1 Hikarigaoka, Fukushima City, Fukushima 960-1295, Japan; Radiation Medical Science Center for the Fukushima Health Management Survey, Fukushima Medical University, 1 Hikarigaoka, Fukushima City, Fukushima 960-1295, Japan; Department of Epidemiology, Fukushima Medical University School of Medicine, 1 Hikarigaoka, Fukushima City, Fukushima 960-1295, Japan; Radiation Medical Science Center for the Fukushima Health Management Survey, Fukushima Medical University, 1 Hikarigaoka, Fukushima City, Fukushima 960-1295, Japan; Department of Epidemiology, Fukushima Medical University School of Medicine, 1 Hikarigaoka, Fukushima City, Fukushima 960-1295, Japan; Radiation Medical Science Center for the Fukushima Health Management Survey, Fukushima Medical University, 1 Hikarigaoka, Fukushima City, Fukushima 960-1295, Japan; Department of Public Health, Fukushima Medical University School of Medicine, 1 Hikarigaoka, Fukushima City, Fukushima 960-1295, Japan; Radiation Medical Science Center for the Fukushima Health Management Survey, Fukushima Medical University, 1 Hikarigaoka, Fukushima City, Fukushima 960-1295, Japan; Radiation Medical Science Center for the Fukushima Health Management Survey, Fukushima Medical University, 1 Hikarigaoka, Fukushima City, Fukushima 960-1295, Japan; Research Institute for Radiation Biology and Medicine, Hiroshima, University, 1-2-3 Kasumi, Minami-ku, Hiroshima City, Hiroshima 734-8553, Japan

**Keywords:** Fukushima, thyroid cancer, nuclear accident, confounding factor, Thyroid Ultrasound Examination, Fukushima Health Management Survey

## Abstract

In response to concerns about health due to radiation exposure, the Fukushima Prefecture launched the Thyroid Ultrasound Examination program for residents aged 0–18 years at the time of the earthquake. Herein, we considered the confounding factors involved in the regional differences in the development of thyroid cancer. In this study, the 242 065 individuals who participated in both first- and second-round surveys were classified into four groups by address according to their air radiation dose. The number of participants diagnosed as malignant or suspicious for malignancy by cytological examination were 17, 38, 10 and 4 with detection rates of 53.8, 27.8, 21.7 and 14.5 per 100 000 participants in Regions 1, 2, 3 and 4, respectively. Sex (*P* = 0.0400), age at the time of the primary examination (*P* < 0.0001) and interval between the first- and second-round surveys (*P* < 0.0001) were significantly different among the four regions, and these were suspected to be confounding factors affecting regional differences in malignant nodule detection rates. In addition, significant regional differences were observed in the participation rate in the confirmatory examination (*P* = 0.0037) and the fine needle aspiration cytology implementation rate (*P* = 0.0037), which could be potential biases. No significant regional differences in the detection of malignant nodules were found in the multivariate logistic regression analysis after adjusting for the survey interval alone or for sex, age and survey interval. The confounding factors and biases identified in this study that may have important impacts on thyroid cancer detection rate should be fully considered in future studies.

## INTRODUCTION

The accident at the Fukushima Daiichi Nuclear Power Plant triggered by the Great East Japan Earthquake in March 2011 caused radioactive contamination and radiation exposure for residents living mainly in the Fukushima Prefecture [1]. Since this accident occurred in multiple reactors, it was evaluated as level 7, equivalent to the Chornobyl Nuclear Power Plant accident.

Childhood thyroid cancer, which was reported after the Chornobyl accident, was a concern in the Fukushima Prefecture; however, the radiation exposure level of Fukushima residents was estimated to be lower than that of Chornobyl residents. The consequences of the accident at Fukushima Daiichi Nuclear Power Plant were much lower than those at Chernobyl (e.g. for the accident in Fukushima, estimated average effective doses to adult evacuees were <6 mSv, and average absorbed doses to the thyroid were <15 mGy, compared with ~30 mSv and 500 mGy for the Chernobyl accident) [2]. To meet the demands of residents and society [3], Fukushima Prefecture has begun a Thyroid Ultrasound Examination (TUE) program for residents under the age of 18 years 7 months after the Fukushima Daiichi Nuclear Power Plant accident [1, 3–5], as part of the Fukushima Health Management Survey (FHMS).

The Preliminary Baseline Survey, also known as the first-round survey of the TUE program, was conducted from October 2011 to March 2014. The first-round survey was conducted during the latent period of the onset of thyroid cancer based on lessons from the atomic bomb survivors and the Chornobyl accident, and 116 participants with thyroid nodules interpreted to be malignant or suspicious for malignancy [6]. Most of the operated cases were diagnosed as papillary thyroid carcinoma [6]. The thyroid cancer cases found in the first round of examination were not evaluated to be due to radiation exposure for the following reasons: (i) no regional differences in thyroid cancer detection rates, (ii) few thyroid cancer cases found in younger age groups and (iii) extremely low radiation doses in participants with thyroid cancer detection [6].

Following the first-round survey, the first Full-Scale Survey, also known as the second-round survey, was conducted from April 2014 to March 2016 [6]. Residents born in Fukushima Prefecture between April 2011 and March 2012 were included in the second-round TUE, and 71 patients with thyroid nodules considered malignant or suspicious for malignancy were found in this round of survey [6]. The characteristics in this round, such as low prevalence of thyroid cancer in the younger age group and extremely low radiation exposure doses in the participants with thyroid cancer, were similar to those in the first-round survey. However, according to the documents from the Fukushima Prefectural Health Management Survey Review Committee, when Fukushima Prefecture was classified into four regions according to air radiation dose, it was pointed out that there may be apparent regional differences in the detection rate of thyroid cancer [7]. Yamamoto *et al*. [8] reported that low-dose radiation exposure after the Fukushima accident correlated with the incidence of thyroid cancer in children. However, this study ignored the confounding factors that may have altered the conclusions.

The TUE program is an observational study that can be classified as a cohort study [5, 6]. In such studies, it is necessary to clarify all confounding factors that may be involved in the data analysis to reach appropriate conclusions [9]. In this report, we identified confounding factors to consider in the analysis of the second-round survey.

## METHODS

### Participants of the TUE program

The subjects for the first-round survey of the TUE program were 367 637 residents of Fukushima Prefecture aged 0–18 years as of 1 April 2011 [6]. The first-round survey was conducted from October 2011 to March 2014, and was extended to April 2015 to provide an opportunity to conduct this examination among nonparticipants. Finally, 300 472 (81.7%) residents participated in this study [6]. The target population for the second-round survey was 381 237 inhabitants, aged 0–19 years on 1 April 2012, in Fukushima Prefecture, including children born in the fiscal year (FY) following the accident (FY2012) [6]. In the second-round survey, all subjects were invited to TUE, including those diagnosed as Grade B or C and thyroid cancer in the first-round survey. The second-round survey was conducted from April 2014 to March 2016 and was completed by 270 552 (71.0%) participants [6]. In this study, the results of the TUE in 242 065 examinees who participated in both first- and second-round surveys according to residential address at the time of the earthquake were analyzed.

### Primary and confirmatory examinations in the TUE program

Both first- and second-round surveys consisted of primary and confirmatory examinations. In the primary examination, all participants underwent ultrasound examinations using a portable apparatus. Thyroid ultrasonography results were classified as Grade A (A1, A2), B or C. Grade B was defined when a nodule larger than 5.1 mm and/or a cyst larger than 20.1 mm in diameter was present. Grade C was defined when a large thyroid nodule or large lymph node metastasis that required immediate further examination was detected. Participants with Grade B or C were recommended to undergo a confirmatory examination.

In the confirmatory examination, ultrasonography was performed using high-performance apparatus, and blood and urine examinations were performed. Fine needle aspiration cytology (FNAC) was recommended for nodules ≥ 5.1 mm in diameter if thyroid carcinoma was strongly suspected, nodules ≥ 10.1 mm in diameter if carcinoma was suspected, all nodules ≥ 20.1 mm in diameter and all cysts ≥ 20.1 mm in diameter [10, 11]. In addition, decision-making regarding whether to receive FNAC depended on the intentions of the examinees and their families. In participants who underwent FNAC in the first-round survey and whose ultrasound examination findings in the second-round survey were similar to those in the first-round survey, repeated FNAC was not recommended.

### Data analysis

**Fig. 1:**
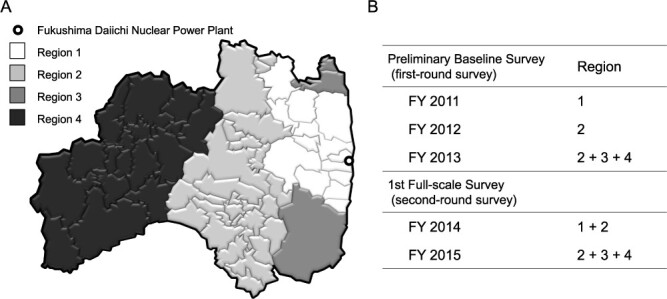
Classification and examination progression by regions in the Fukushima Prefecture. FY: fiscal year.

In this study, a data set fixed on 31 March 2020 was used. The participants of this study were classified into 59 municipalities in Fukushima Prefecture according to their addresses at the time of the earthquake. They were then classified into four regions according to the air dose in the year following the earthquake and administrative districts (Fig. 1). The first-round survey for Region 1 and a part of Region 2 was conducted in FY 2011 and 2012, respectively, whereas the examinations for Regions 3 and 4, and the rest of Region 2 were conducted in FY 2013. The second-round survey was conducted in Region 1 and a part of Region 2 in FY 2014, and the survey in FY 2015 was performed in Regions 3 and 4, and the remaining parts of Region 2 (Fig. 1). All statistical analyses were performed using SAS 9.4 (SAS Institute Inc, Cary, NC, USA). Statistical significance was set at *P* < 0.05.

### Study ethics

This study was approved by the Ethics Review Committee of Fukushima Medical University (No. 1318). Informed consent was obtained from the parents or guardians of all the surveyed children in accordance with the ethical guidelines at the time the examinations were conducted (FYs 2014–2015). Examinees aged ≥ 20 years provided consent themselves. The raw data used to create all tables in the present study were unavailable because of a restriction outlined in the informed consent form.

## RESULTS

### Regional differences in results from the second-round survey and confounding factors

The regional differences in the detection rate of malignancy in the second-round survey were evaluated among 242 065 examinees who participated in both first- and second-round surveys whose address information at the time of the earthquake was available. In the second-round survey, 71 examinees were diagnosed as malignant or suspicious for malignancy by cytological examination, whereas the number of cases with malignant nodules was 69 in subjects of this study who participated in both the first- and second-round surveys. Of these, 57 cases had no nodules in the first-round survey. The participants were divided into four groups according to their address at the time of the earthquake and were analyzed (Table 1). The number of participants diagnosed as malignant or suspicious for malignancy by cytological examination was 17, 38, 10 and 4, with detection rates of 53.8, 27.8, 21.7 and 14.5 per 100 000 participants in Regions 1–4, respectively. There was a significant difference in the detection rate of malignancy (*P* = 0.0204) and thyroid nodules (*P* < 0.0001) among the four regions, and the highest rates for both cases were observed in Region 1.

**Table 1:** Characteristics of subjects participated to the second-round surveys in each region

	Region 1	Region 2	Region 3	Region 4	Total	*P* value
Participants, *n*	31 577	136 791	46 030	27 667	242 065	
Sex (female), %	50.3	49.5	50.0	49.7	49.8	0.0400^d^
Second-round survey						
Age at the primary examination Median (25–75%) Mean (SD)	12 (8–16) 9.3 (4.7)	12 (8–15) 9.8 (4.6)	13 (9–16) 10.4 (4.5)	12 (9–15) 10.4 (4.1)	12 (8–16) 9.9 (4.6)	<0.0001^e^
Participants with nodules, *n* (%)	561 (1.78)	1924 (1.41)	614 (1.33)	363 (1.31)	3462 (1.43)	<0.0001^d^
<10.1 mm, *n* (%)	487 (1.54)	1615 (1.18)	493 (1.07)	299 (1.08)	2894 (1.20)	<0.0001^d^
≥10.1 mm, *n* (%)	74 (0.23)	309 (0.23)	121 (0.26)	64 (0.23)	568 (0.23)	0.5667^d^
Participants with Grade B or C, *n* (%)	325 (1.03)	1102 (0.81)	378 (0.82)	222 (0.80)	2027 (0.84)	0.0010^d^
Participants who completed the confirmatory examination, *n* (%)^a^	282 (86.8)	910 (82.6)	327 (86.5)	173 (77.9)	1692 (83.5)	0.0136^d^
Participants examined with FNAC, *n* (%)^b^	37 (13.1)	117 (12.9)	26 (8.0)	9 (5.2)	189 (11.2)	0.0040^d^
Participants with malignant nodules, *n*^c^	17	38	10	4	69	0.0204^d^
Detection rate of malignant nodules, per 100 000 persons	53.8	27.8	21.7	14.5	28.5	
Interval from the first-round to the second-round survey (years), median (25–75%)	2.41 (2.31–2.62)	2.07 (1.99–2.15)	2.14 (2.03–2.37)	1.94 (1.82–2.01)	2.09 (1.99–2.25)	<0.0001^e^

^a^Participants who completed the confirmatory examination/participants with thyroid nodules subjected to the confirmatory examination.

^b^Participants examined with FNAC/participants with thyroid nodules subjected to the confirmatory examination,

^c^Participants with thyroid nodules interpreted as malignant or suspicious for malignancy

^d^Chi-square test

^e^Kruskal–Wallis test

To explore confounding factors, we analyzed factors that might have affected the results of the second-round survey. There were significant differences among the four regions of residence during the disaster by sex (*P* = 0.0400), age at the time of the primary examination (*P* < 0.0001), participation rate of the secondary confirmatory examination (*P* = 0.0037) and FNAC implementation rate (*P* = 0.0037).

### Association of survey interval with the second-round survey results

The first-round survey was conducted in the order of Regions 1–4 over a 2.5-year period, and the second-round survey was conducted in the same order over a 2-year period (Fig. 1). This resulted in significant regional differences in survey intervals (*P* < 0.0001), and the longest interval was observed in Region 1, which had the highest detection rate of malignant nodules in the second-round survey (Table 1).

According to the classification of survey interval (<2, 2–2.5 and ≥3 years) as shown in Table 2, the regions with higher proportions of participants were Regions 2 and 4 in the <2 years group, Region 2 in the 2–2.5 years group and Regions 1 and 3 in the ≥3 years group. The detection rates of nodules diagnosed as malignant or suspicious for malignancy per 100 000 examinees were 13.0, 29.7 and 65.0, in ascending order of the survey interval. In addition, the detection rate of nodules and the rate of Grade B or C were highest in the group with the longest interval. These results indicated that survey interval was a confounding factor in the analysis of regional differences in thyroid cancer detection.

**Table 2:** Characteristics of the participants of the first- and second-round surveys in groups categorized by survey interval

		<2 years	≥2, <2.5 years	≥3 years	*P* value
Participants, *n*		69 192	148 273	24 600	
Sex (female), %		49.7	49.6	50.7	0.0075^d^
Second-round survey					
	Age at the primary examination Median (25–75%)	12 (8–16)	12 (9–15)	14 (10–17)	*<*0.0001^e^
	Participants with nodules, *n* (%)	994 (1.44)	1986 (1.34)	482 (1.96)	<0.0001^d^
	<10.1 mm, *n* (%)	831 (1.20)	1659 (1.12)	404 (1.64)	<0.0001^d^
	≥10.1 mm, *n* (%)	163 (0.24)	327 (0.22)	78 (0.32)	0.0150^d^
	Participants with Grade B or C, *n* (%)	571 (0.83)	1156 0.78)	300 (1.22)	<0.0001^d^
	Participants who completed the confirmatory examination, *n* (%)^a^	455 (79.7)	989 (85.6)	248 (82.7)	0.0078^d^
	Participants examined with FNAC, *n* (%)^b^	37 (8.1)	121 (12.2)	31 (12.5)	0.0549^d^
	Participants with malignant nodules, *n*^c^	9	44	16	0.0002^d^
	Detection rate of malignant nodules, per 100 000 persons	13.0	29.7	65.0	
Region of residence during the 2011 disaster, (%)	Region 1	2.9	12.7	44.0	*P* < 0.0001^e^
	Region 2	53.9	63.4	22.1	
	Region 3	13.8	19.0	33.7	
	Region 4	29.3	4.9	0.23	

^a^Participants who completed the confirmatory examination/participants with thyroid nodules were subjected to the confirmatory examination.

^b^Participants examined with FNAC/participants who had nodules ≥ 5.1 mm confirmed in the confirmatory examination.

^c^Participants with thyroid nodules interpreted as malignant or suspicious for malignancy.

^d^Chi-square test

^e^Kruskal–Wallis test

### Regional differences in adjusted odds ratios of malignant thyroid nodules

As shown in Table 3, logistic regression analysis was performed to investigate regional differences in the detection of malignant thyroid nodules in Fukushima Prefecture. The unadjusted odds ratio (OR), with Region 4 as a reference, was significantly higher in Region 1 at 3.73 [95% confidence intervals (CIs): 1.25–11.07], whereas Regions 2 and 3 exhibited no significant increase in OR. Multiple logistic regression analyses were then performed to adjust for confounding factors. The results after adjusting for sex or age at the primary examination were similar to those for unadjusted ORs; however, after adjusting for survey interval, the OR in Region 1 did not increase significantly to 2.43 (0.75–7.91). Furthermore, there was no significant increase in the OR for Region 4 after adjusting for sex, age and interval.

**Table 3:** OR of nodules diagnosed as malignant or suspicious for malignancy according to regional groups

		OR (95% CI)			
		Region 1	Region 2	Region 3	Region 4
Malignant nodule (*n* = 69)		3.73 (1.25–11.07)	1.92 (0.69–5.38)	1.50 (0.47–4.79)	Ref.
Adjusted for					
	A. Sex	3.72 (1.25–11.06)	1.92 (0.69–5.39)	1.50 (0.47–4.79)	Ref.
	B. Age at primary examination	3.67 (1.23–10.90)	1.89 (0.68–5.31)	1.27 (0.40–4.05)	Ref.
	C. Survey interval	2.43 (0.75–7.91)	1.69 (0.60–4.76)	1.23 (0.38–3.99)	Ref.
	A, B and C	3.22 (0.997–10.37)	1.82 (0.65–5.15)	1.21 (0.38–3.91)	Ref.

ORs and 95% CIs were calculated by logistic regression analyses.

^a^Participants with thyroid nodules diagnosed as malignant or suspicious for malignancy.

In the participants of this program, the malignant nodule detection rate could be influenced by the decision of participants or their family members at each stage of this examination. Notably, the decision to participate in the confirmatory examination could be biased in the results. Therefore, we examined the regional ORs for malignant nodule among those who underwent confirmatory examinations under conditions that excluded regional differences in the participation rate of confirmatory examination (Table 4). The results showed that Regions 1–3 tended to have higher ORs than Region 4, but there was no significant increase in either unadjusted or adjusted ORs. Moreover, ORs in the participants of the confirmatory examination were lower than the ORs in the primary examination participants.

**Table 4:** OR of malignant nodule in the participants of confirmatory examination according to regional groups

		Region 1	Region 2	Region 3	Region 4
Participants who completed the confirmatory examination, *n*		282	910	327	173
Participants with malignant nodules^a^, *n* (%)		17	38	10	4
OR of malignant nodule (*n* = 69)		2.71 (0.90–8.19)	1.84 (0.65–5.23)	1.33 (0.41–4.31)	Ref.
Adjusted OR for					
	A. Sex	2.66 (0.88–8.05)	1.78 (0.63–5.06)	1.28 (0.39–4.14)	Ref.
	B. Age at primary examination	2.80 (0.93–8.48)	1.88 (0.66–5.34)	1.33 (0.41–4.31)	Ref.
	C. Survey interval	2.10 (0.65–6.84)	1.70 (0.59–4.86)	1.21 (0.37–3.96)	Ref.
	A, B and C	2.27 (0.69–7.44)	1.72 (0.60–4.93)	1.20 (0.37–3.92)	Ref.

ORs and 95% CIs of malignant nodule in participants who completed the confirmatory examination were calculated by logistic regression analyses.

^a^Participants with thyroid nodules interpreted as malignant or suspicious for malignancy.

## DISCUSSION

Assessing the association between the occurrence of thyroid cancer and low-dose radiation exposure using TUE results is an important public health issue. As one of the analyses for this purpose, regional differences in results of the TUE conducted for 2 years from the 4th year after the accident were investigated in this study. The regions in this study were classified as follows: Region 1 consists of the municipalities, including the Fukushima Daiichi Nuclear Power Plant and the areas designated as evacuation zones after the nuclear accident; Region 2 is the Nakadori area, which is located in the middle of Fukushima Prefecture and has the largest population; Region 3 consists of the municipalities on the Pacific coast called the Hamadori area excluding the evacuation areas; and Region 4 is the Aizu district in the western area of Fukushima Prefecture, which is the farthest from the Fukushima Daiichi Nuclear Power Plant. The estimated air radiation doses were in the order of Regions 1 > 2 > 3 > 4 [10, 12]. The maximum dose in Regions 1–4 of average estimated effective doses to 1-year-old infants in the first year after the accident by the municipality was 5.4, 4.3, 1.7 and 1.3 mSv, respectively, and the highest estimated external doses were found in Regions 1 and 2, followed by Region 3, and the lowest doses were observed in Region 4 [13, 14]. The maximum dose in Regions 1–4 of average estimated absorbed doses to the thyroids of 1-year-old infants by municipality were 30, 15, 10 and 3.7 mSv, respectively, and the estimated absorbed doses in the four regions were considered to be Regions 1 > 2 > 3 > 4 [13, 15].

In the present study, regional differences in thyroid cancer detection in Fukushima Prefecture were analyzed. For a scientifically accurate analysis, it is important to consider confounding factors that affect the detection rate of thyroid cancer. In addition to radiation exposure, various other factors such as sex and age are known to be involved in the development of thyroid cancer [16, 17]. According to cancer statistics in Japan in 2019, the incidence rate of thyroid cancer per 100 000 individuals for all ages was 8.0 cases in males and 21.5 cases in females, with females being affected 2.7 times more than males [18]. The sex disparity in the detection rate of thyroid cancer was also evident in adolescents and young adults [19]. An age-dependent increase in thyroid cancer detection rate has been reported in the national cancer registry in Japan, even in Fukushima after the nuclear accident [20]. In our TUE program, the detection rate of malignant nodules was higher in females, and an increase in the detection rate with increasing age was also reported. [6, 21]. In this study, there was a significant regional difference in the male-to-female ratio of the examinees (Table 1). In addition, in each round of the TUE, examination was started in Region 1, then carried over to a part of Region 2, and finally implemented in the remaining area of Region 2 and in Regions 3 and 4 in the following year. Therefore, age at the time of examination was lowest in Region 1 and highest in Regions 3 and 4 (Table 1). This clearly indicated that sex and age are confounding factors that should be adjusted for when examining regional differences in the detection rate of malignant nodules. However, even when the sex or the age at the time of primary examination was adjusted, there was a significant difference in the detection rate of malignant nodules among regions.

In the second-round survey, 69 nodules diagnosed as malignant or suspicious for malignancy were newly identified after a median interval of 2.09 years from the first-round survey (Table 1). Due to the TUE schedule, there were significant differences in interval among regions, with Region 1 having the longest median interval of 2.41 years and Region 4 having the shortest median interval of 1.94 years. Of the 69 participants with malignant nodules diagnosed in the second-round survey, 57 cases did not even have a nodule on the first-round survey, clearly indicating that the survey interval since the previous examination affects the number of malignant nodules detected. Therefore, it was suggested that the interval between examinations was an important confounding factor that affected the results of the second-round survey. In this study, no statistically significant regional differences in OR for the detection of malignant nodules were observed after adjusting for either the examination interval alone or the combination of the interval, sex and age. These results indicate that confounding factors should be fully considered when assessing the effects of radiation exposure by analyzing regional differences. Yamamoto *et al*. [8] reported that low-dose radiation exposure after the Fukushima accident correlated with the incidence of thyroid cancer in children. However, this study did not consider the confounding factors that may have altered the conclusions.

The present study has several limitations. In addition to confounders such as sex, age and interval of examination, an additional bias would be the participation rate of the confirmatory examination. Among the participants in this study, 16.5% of examinees who had nodules ≥ 5.1 mm or cysts ≥ 20.1 mm for which a confirmatory examination was recommended did not undergo the confirmatory examination in the second-round survey (Table 1). Although the TUE program has the characteristics of an epidemiological study, its most important purpose is to support the health and welfare of the residents of Fukushima Prefecture. Therefore, TUE results are affected by the rate of participation, which is decided by the voluntary intention of local residents [6, 22]. Even in this study, which was a study of primary examination participants, the regional differences in the rate of confirmatory examination, which was highest in Region 1 and lowest in Region 4, had a direct impact on the rate of thyroid cancer detection. Because these data cannot be treated as individual data, regional differences in participation rates for confirmatory examination cannot be incorporated into the OR adjustment. Therefore, we examined regional differences in the detection of malignant nodules among the examinees of confirmatory examination, which eliminated the influence of the participation rate of the confirmatory examination. The ORs among those who underwent confirmatory examination were lower than those who participated in the primary examination, with no significant increase in ORs in Regions 1–3. These results might indicate that the participation rate of confirmatory examination was a bias that can affect regional differences in the detection rate of malignant nodules.

Second, there were regional differences in the implementation rates of FNAC. In the TUE, FNAC was not performed on every nodule found but in a limited number of participants according to the guidelines published by Japanese academic societies [10, 11, 23]. In fact, FNAC was performed in 11.2% of examinees with nodules who were eligible for the confirmatory examination, and this implementation rate was highest in Region 1 (13.1%) and decreased in the order of Regions 2–4, with the lowest rate in Region 4 (5.2%) (Table 1). Since the implementation of FNAC was judged based on nodule size and ultrasound findings [11, 23], the possibility that there were regional differences in nodular size and ultrasound findings of malignancy cannot be ruled out. However, individuals’ wishes were also considered in the decision to implement FNAC based on the context of the TUE program supporting the health and well-being of the population. Therefore, to accurately interpret the TUE results, it is necessary to recognize the possibility that the voluntary decisions of the examinees and their families in conducting the FNAC may have influenced the results.

The third limitation of this study was that the relationship between regions in Fukushima Prefecture and exposed radiation dose might not be rigidly high. In particular, it was estimated that the absorbed thyroid doses among residents in evacuation zones vary greatly, depending on the evacuation route after the accident [2]. Therefore, the analysis of thyroid cancer detection rates by regional differences might be insufficient to analyze the effects of radiation exposure. Recently, a method was developed to estimate individual thyroid equivalent doses from internal radionuclides based on post-accident behavioral records [24], and future analyses using individual radiation doses are expected.

In the present study, no significant regional difference was found in the detection rate of malignant nodules in the second-round survey after adjustment for confounding factors that were associated with thyroid cancer development and regional differences. However, the OR of thyroid cancer detection tended to be higher in areas with higher air dose. The present analysis suggests the possibility that factors that cannot be included in the adjustment factors may be considered as bias factors. Particularly, the confirmatory examination participation rate was found to have an effect as a bias since the OR was found to be lower in the analysis of those who underwent the confirmatory examination. Furthermore, it was also suggested that the rate of FNAC implementation could also be considered as a bias. These biases may be due to ecological biases such as regional differences in health concerns about radiation exposure. Such ecological biases could be serious limitations in the analysis of this examination. In fact, in the first-round survey of this TUE, it was reported that those who lived in the western part of the prefecture, i.e. Region 4, at the time of the earthquake were more likely to have not undergone an examination [22]. Although it is necessary to include the results of the next and subsequent rounds of survey to statistically verify whether there are regional differences in thyroid cancer detection rates, the confounding factors and biases that have an important impact on thyroid cancer detection rates should be fully considered in future studies.

## CONFLICT OF INTEREST

No potential conflict of interest relevant to this article was reported.

## FUNDING

This work was supported by the National Health Fund for Children and Adults Affected by the Nuclear Incident that facilitated the efficient design and conduction of our study.

## DATA AVAILABILITY

The data used in this paper were obtained by the Fukushima Health Management Survey; the original data are not generally available.
